# Prevalence of stunting among under-five children in refugee and internally displaced communities: a systematic review and meta-analysis

**DOI:** 10.3389/fpubh.2023.1278343

**Published:** 2023-11-29

**Authors:** Priyanka Choudhary, Bijaya K. Padhi, Amit Kumar Mital, Aravind P. Gandhi, Sanjeeb Kumar Mishra, Neha Suri, Sudhansu Sekhar Baral, Prakasini Satapathy, Muhammad Aaqib Shamim, Lakshmi Thangavelu, Sarvesh Rustagi, Ranjit Sah, Mahalaqua Nazli Khatib, Shilpa Gaidhane, Quazi Syed Zahiruddin, Alaa Abd-Alrazaq, Hashem Abu Serhan

**Affiliations:** ^1^Department of Community Medicine, Shri Atal Bihari Vajpayee Government Medical College, Faridabad, India; ^2^Department of Community Medicine and School of Public Health, Postgraduate Institute of Medical Education and Research, Chandigarh, India; ^3^Department of Paediatrics, Shri Atal Bihari Vajpayee Government Medical College, Faridabad, India; ^4^Department of Community Medicine, All India Institute of Medical Sciences, Nagpur, India; ^5^Department of Community Medicine, Veer Surendra Sai Institute of Medical Science and Research (VIMSAR), Sambalpur, Odisha, India; ^6^Department of Physical Medicine and Rehabilitation, Post Graduate Institute of Medical Education and Research, Chandigarh, India; ^7^School of Pharmacy, Graphic Era Hill University, Dehradun, India; ^8^Evidence Synthesis Lab, Kolkata, India; ^9^Department of Pharmacology, All India Institute of Medical Sciences, Jodhpur, India; ^10^Center for Global Health Research, Saveetha Medical College and Hospital, Saveetha Institute of Medical and Technical Sciences, Saveetha University, Chennai, India; ^11^School of Applied and Life Sciences, Uttaranchal University, Dehradun, Uttarakhand, India; ^12^Tribhuvan University Teaching Hospital, Kathmandu, Nepal; ^13^Department of Clinical Microbiology, DY Patil Medical College, Hospital and Research Centre, DY Patil Vidyapeeth, Pune, Maharashtra, India; ^14^Division of Evidence Synthesis, Global Consortium of Public Health and Research, Datta Meghe Institute of Higher Education, Wardha, India; ^15^One Health Centre (COHERD), Jawaharlal Nehru Medical College, Datta Meghe Institute of Higher Education, Wardha, India; ^16^Global Health Academy, Division of Evidence Synthesis, School of Epidemiology and Public Health and Research, Jawaharlal Nehru Medical College, Datta Meghe Institute of Higher Education and Research, Wardha, India; ^17^AI Center for Precision Health, Weill Cornell Medicine, Doha, Qatar; ^18^Department of Ophthalmology, Hamad Medical Corporation, Doha, Qatar

**Keywords:** under five children, refugee, internally displaced person, sustainable developmental goals, stunting

## Abstract

**Background:**

A pooled estimate of stunting prevalence in refugee and internally displaced under-five children can help quantify the problem and focus on the nutritional needs of these marginalized groups. We aimed to assess the pooled prevalence of stunting in refugees and internally displaced under-five children from different parts of the globe.

**Methods:**

In this systematic review and meta-analysis, seven databases (Cochrane, EBSCOHost, EMBASE, ProQuest, PubMed, Scopus, and Web of Science) along with “preprint servers” were searched systematically from the earliest available date to 14 February 2023. Refugee and internally displaced (IDP) under-five children were included, and study quality was assessed using “National Heart, Lung, and Blood Institute (NHLBI)” tools.

**Results:**

A total of 776 abstracts (PubMed = 208, Scopus = 192, Cochrane = 1, Web of Science = 27, Embase = 8, EBSCOHost = 123, ProQuest = 5, Google Scholar = 209, and Preprints = 3) were retrieved, duplicates removed, and screened, among which 30 studies were found eligible for qualitative and quantitative synthesis. The pooled prevalence of stunting was 26% [95% confidence interval (CI): 21–31]. Heterogeneity was high (*I*^2^ = 99%, *p* < 0.01). A subgroup analysis of the type of study subjects revealed a pooled stunting prevalence of 37% (95% CI: 23–53) in internally displaced populations and 22% (95% CI: 18–28) among refugee children. Based on geographical distribution, the stunting was 32% (95% CI: 24–40) in the African region, 34% (95% CI: 24–46) in the South-East Asian region, and 14% (95% CI: 11–19) in Eastern Mediterranean region.

**Conclusion:**

The stunting rate is more in the internally displaced population than the refugee population and more in the South-East Asian and African regions. Our recommendation is to conduct further research to evaluate the determinants of undernutrition among under-five children of refugees and internally displaced populations from different regions so that international organizations and responsible stakeholders of that region can take effective remedial actions.

**Systematic review registration:**

https://www.crd.york.ac.uk/prospero/display_record.php?RecordID=387156, PROSPERO [CRD42023387156].

## Introduction

Stunting has been defined as the “height-for-age *z*-score of more than two standard deviations below the World Health Organization (WHO) Child Growth Standards median,” (1) which depicts the restriction of a child's potential growth (2). Globally, in 2019, 21.3% or 144 million under-five children were stunted, i.e., low height-for-age (3). In 2019, Asia represented more than half of all under-five stunted children (54%, 78.2 million) and two out of five under-five stunted children lived in Africa (40%, 57.5 million), and 4.7 million lived in Latin American and Caribbean regions (1). Stunting among children has been reported to exceed 30% in eastern Africa (34.5%), middle Africa (31.5%), southern Asia (31.7%), and Oceania (38.4%), excluding Australia and New Zealand. The long-term consequences of stunted children are shorter adult height, more susceptibility to chronic diseases in adulthood, reduced attained schooling rate, and less adult income (4).

The United Nations High Commissioner for Refugees (UNHCR) has defined a refugee as “someone who has been forced to flee his or her country because of persecution, war, or violence. A refugee has a well-founded fear of persecution for various reasons of race, religion, nationality, political opinion, or membership in a particular social group” (5). Just five countries contribute to 69% of the displaced population across borders, i.e., Syria, Venezuela, Afghanistan, South Sudan, and Myanmar. According to UNHCR's Global Report 2021, 89.3 million people worldwide were refugees (6). Internally displaced persons (IDP) are “those who has[sic] been forced to flee their home due to internal strife and natural disasters but has never crossed an international border. These persons seek safety anywhere in nearby towns, schools, settlements, internal camps, even forests, and fields.” These people are the largest group that UNHCR assists (5). Countries such as Yemen, Colombia, Syria, and the Democratic Republic of the Congo contribute to the largest internally displaced populations globally. In the year 2021, 53.2 million people were internally displaced around the world (6). In the country of origin of refugees, children are vulnerable to vaccine-preventable diseases, dental problems, nutritional deficiencies, chronic infections, and non-communicable diseases due to lack of accessibility to health care in conflict areas for a prolonged period (7–9). During their journey to another country, children are at risk of communicable diseases such as diarrhea, respiratory infections, skin infections, and others due to inadequate hygiene and sanitation facilities (10). In the country of destination, the refugee groups are most vulnerable to acute food insecurity and malnutrition (11, 12). Chronic undernutrition is very common in refugees and internally displaced populations, with a prevalence of 9–54% (13–16). Pooled estimates of stunting prevalence in refugee and internally displaced communities can assist in quantifying the problem and global resource mobilization toward that problem. Previous systematic reviews on the undernutrition among the under-five children of refugees and migrant populations reported a prevalence ranging up to 23.8% (17, 18). A similar review on chronic undernutrition status among the under-five children of IDP could not be found although the circumstances leading to refugee and IDP conditions overlap. Due to the paucity of pooled data on stunting in this population group, the objective of this study was to estimate the pooled prevalence of stunting in refugees as well as internally displaced children aged < 5 years from different parts of the globe.

## Methods

The present systematic review and meta-analysis (SRMA) was conducted adhering to the PRISMA guidelines (19) (Supplementary Table 1).

### Participants

The study participants were refugees and internally displaced children who were ≤ 5 years. The participation of children in this study was not limited to gender, social status, or ethnicity. A refugee is “a person who is outside his habitual residence or country of nationality due to fear of persecution because of his race, religion, nationality, membership and is unable or unwilling to avail himself of the protection of that country, or to return there, for fear of persecution” (20). An internally displaced person (IPD) is one who has been forced to flee their home due to internal strife and natural disasters but has never crossed an international border (21).

### Eligibility criteria

Population Intervention Comparator and Outcome (PICO) criteria were used to search the research question, “What is the prevalence of stunting among under-five children in refugee and internally displaced communities?” All studies that reported stunting in under five refugees or IDPs were eligible, irrespective of publication year. The full-text articles written other than English language were not considered for this review as the research team could not search for, retrieve, and translate literature published in other languages due to lack of logistics and financial support (Table 1).

**Table 1:** Inclusion and exclusion criteria.

**Research question: What is the prevalence of stunting among under five children in refugee and internally displaced communities?**		
	**Inclusion**	**Exclusion**
Participants	Refugee/internally displaced person Under-five children • All genders are included	Non-pediatric cases Migrant children
Disease	Stunting	Wasting, underweight, overweight, obesity, and micronutrient deficiency Non-communicable diseases due to overnutrition and undernutrition
Outcome	Prevalence of stunting	Risk factors/determinants of malnutrition, i.e., overweight, obesity, stunting, wasting, underweight, and micronutrient deficiency. Hospitalization Mortality due to malnutrition
Study designs	Prevalence studies, cross-sectional studies, case–control studies, cohort studies	Qualitative, policy, opinions, case studies, case series, case reports, and randomized control trial
	Geography: Global level Date of search: Publish till February 2023 English language Human studies Published and unpublished data	

### Search strategy and selection criteria

Suitable search terms and Boolean operators (“AND,” “OR,” and “NOT”) were used to conduct the comprehensive search from the seven electronic bibliographic databases: Cochrane, EBSCOHost-Academic Search Complete, EMBASE, ProQuest, PubMed, Scopus, and Web of science. Preprint servers such as medRxiv, arXiv, bioRxiv, BioRN, ChiRxiv, ChiRN, and SSRN were incorporated as search databases (Supplementary Table 2). The following combination of search terms and keywords was used in the search:

Refugee^*^ OR expat^*^ OR asylum seeker^*^ OR displaced person^*^
AND
Malnutrition OR undernutrition OR undernourish OR stunting OR stunt^*^
AND
Under five child^*^ OR preschool child^*^ OR less than 2 year^*^ OR preschool child.

The retrieved studies from various databases were imported into Mendeley Desktop V1.19.5 software to coordinate the review process, remove duplicates, and manage citations. The title/abstract of the retrieved studies was screened for eligibility, and further full text of the eligible studies was appraised. The articles that met the inclusion criteria were kept for data extraction. The first search strategy was implemented in December 2022. Prior to the final analysis, the strategy was re-run in February 2023. The study was registered in PROSPERO with registration number CRD42023387156.

### Data extraction and management

**Figure 1:**
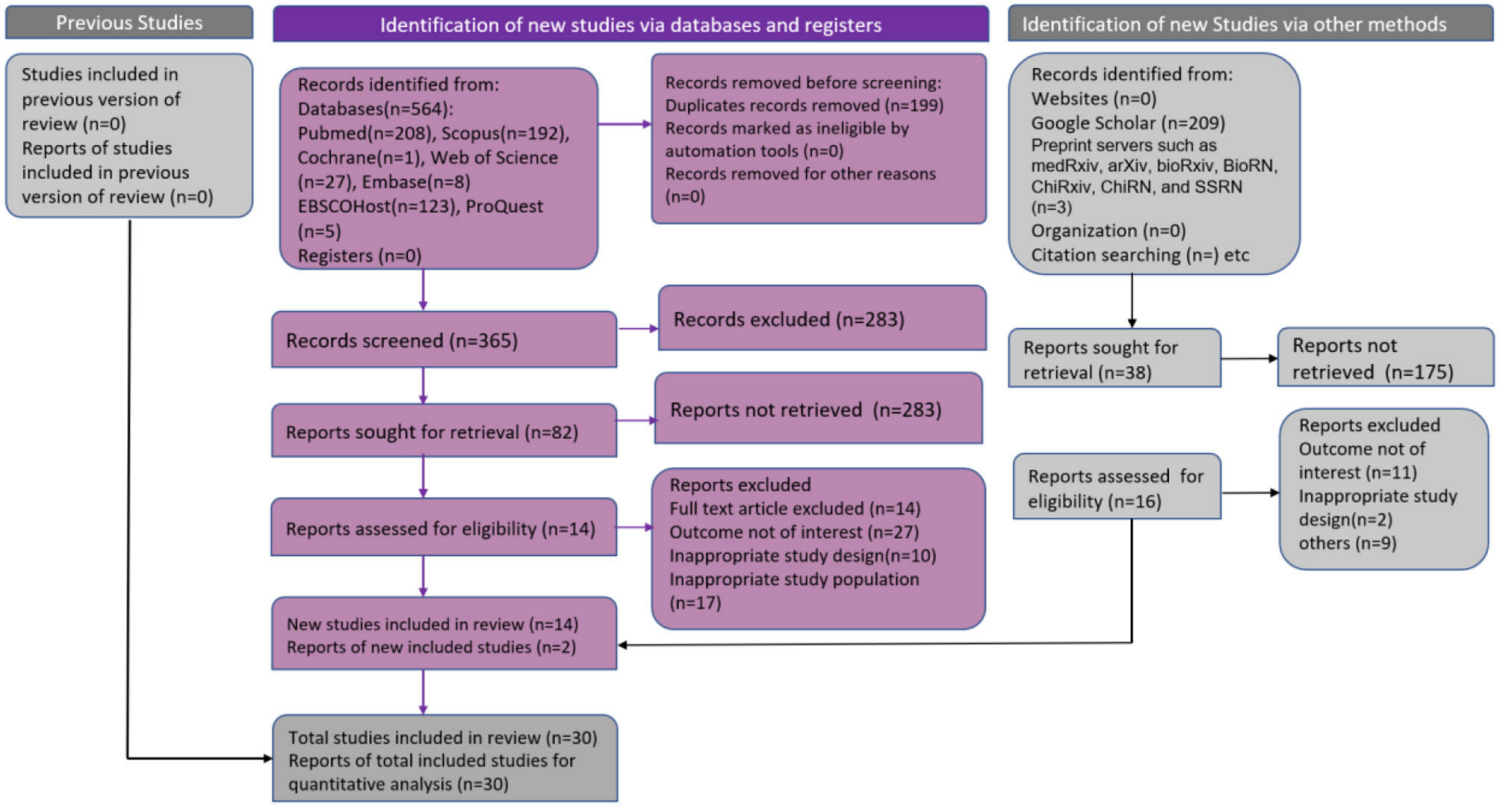
PRISMA flow chart. Flow chart showing included studies in systematic review and meta-analysis of stunting in refugee/internally displaced children.

Two independent authors (PC and SM) independently conducted the entire review screening process; any disagreement about including a study for full-text review was resolved through discussion and consensus. Further consultation was done with the third co-author (AGP) to assess the title abstracts if there was still disagreement between the two co-authors about the inclusion of any study in this analysis. The third co-author would decide whether to include the study in a full-text review. Data extraction from the eligible full-text articles was done by two authors (PC and SM) independently. At the end of the independent extraction, a meeting was conducted to remove the discrepancies in data extraction between the authors. The third author (AGP) settled conclusively the irresolvable contradictions. Microsoft Excel spreadsheet was utilized, and a data extraction table was formulated. From each of the final eligible studies, the following information was gathered: the author's name, the year of publication, the place of study, the origin country of refugee/IPDs, the study design, the number of participated children, prevalence of stunting in under-five children, age of included children, and gender-wise distribution of children. The Preferred Reporting Standard of Systematic Reviews and Meta-Analysis (PRISMA) flow-chart and PRISMA 2020 (Preferred Reporting Items for Systematic Reviews and Meta-Analyses) checklist were used to ensure the scientific precision of the searched articles (Figure 1; Supplementary Table 1).

### Quality assessment

In order to assess the quality of the eligible observational cohort and cross-sectional studies, study assessment tools with fourteen criteria checklists from the National Heart, Lung, and Blood Institute (NHLBI) were used (22). Studies that met ten to fourteen criteria were considered good quality studies, with five to nine criteria considered fair and four or less criteria considered poor. A high rating implies a low risk of bias, and a low rating implies a high risk of bias (22).

### Data analysis

The extracted data were imported into R Studio Software. A descriptive statistic of the selected studies is depicted in tables and figures. The pooled estimate of stunting was determined using a random-effects model (Dersimonian–Laird method). The studies retrieved are expected to be heterogeneous because of different geographical study areas, sample sizes, study designs, age of study participants, study periods, and methodology (23–25). The statistical heterogeneity was checked by forest plot and *I*^2^ statistics (24). The outliers in the study were identified by a Baujat plot and diagnostic plot followed by a leave-one-out meta-analysis. Contour-enhanced funnel plot, Doi plot, LFK index (26), and Egger statistics were used to evaluate the publication bias (small study effect). Sensitivity analysis was performed by removing low-quality studies, and then the pooled estimate was determined. Graphical display of study heterogeneity (GOSH) plot analysis [*K*-means, Density-Based Spatial Clustering of Applications with Noise (DBSCAN), Gaussian] was also undertaken to identify the outliers, and a pooled estimate was arrived at after removing all the outliers, simultaneously. Heterogeneity was explored by means of subgroup analysis (27) according to the type of population (refugee & IDPs) and geographical origin of the refugees. The mixed effect model has been used for subgroup analysis. The studies within a subgroup are pooled using a common-effects model, and the subgroups themselves are pooled using a random-effect model as these subgroups might differ from each other. Here, the dependent variable is the stunting rate, and the variables based on which subgroups are made are the variables that might impact the effect size of the dependent variable. All analyses were conducted in R Studio following the standard codes (28).

### Ethics

Since systematic review and meta-analysis were conducted with published literature data, ethical permission was not required.

## Results

### Search and screening results

A total of 776 articles were yielded in the systematic search from different databases such as Cochrane (1), EBSCOHost-Academic Search Complete (123), EMBASE (8), ProQuest (5), PubMed (208), Scopus (192), Web of science (27), preprint servers such as medRxiv, arXiv, bioRxiv, BioRN, ChiRxiv, ChiRN and SSRN (3), and Google Scholar (209). Among them, 199 duplicate studies were excluded. The title/abstract screening of 577 articles was done, and 458 articles were removed due to ineligibility. Full-text screening was performed on 120 eligible articles. Among them, 90 articles were eliminated as they did not satisfy the inclusion criteria. In total, 30 studies were eligible and included in the systematic review and meta-analysis. The process has been demonstrated in the PRISMA flow (Figure 1).

#### Quality assessment

The quality assessment of the included study findings is demonstrated in Table 2. Of the 30 studies, 14 (46.7%) were found to be of good quality and 16 (53.3%) of fair quality.

**Table 2:** Quality assessment of included cross-sectional studies with the use of NIH quality assessment tool.

**References**	**Q1**	**Q2**	**Q3**	**Q4**	**Q5**	**Q6**	**Q7**	**Q8**	**Q9**	**Q10**	**Q11**	**Q12**	**Q13**	**Q14**	**Quality rating**
Abdeen et al. (29)	Y	Y	Y	Y	Y	NA	NA	NA	NA	NA	Y	N	NA	NA	Good
Abou-Rizk et al. (30)	Y	Y	Y	Y	Y	NA	NA	NA	NA	NA	Y	N	NA	NA	Good
Abukishk et al. (31)	Y	Y	Y	Y	NI	NA	NA	NA	NA	NA	Y	N	NA	NA	Fair
Akeh et al. (32)	Y	Y	Y	Y	Y	NA	NA	NA	NA	NA	Y	N	NA	NA	Good
Ali et al. (14)	Y	Y	Y	Y	Y	NA	NA	NA	NA	NA	Y	N	NA	NA	Good
Bilukha et al. (33)	Y	Y	Y	Y	NI	NA	NA	NA	NA	NA	Y	N	NA	NA	Fair
Bougma et al. (34)	Y	Y	Y	Y	CD	NA	NA	NA	NA	NA	Y	N	NA	NA	Fair
Brhane et al. (35)	Y	Y	Y	Y	Y	NA	NA	NA	NA	NA	N	N	NA	NA	Fair
Ejigu et al. (36)	Y	Y	Y	Y	Y	NA	NA	NA	NA	NA	Y	N	NA	NA	Good
El Kishawi et al. (16)	Y	Y	Y	Y	CD	NA	NA	NA	NA	NA	Y	N	NA	NA	Fair
Faine et al. (37)	Y	Y	Y	Y	Y	NA	NA	NA	NA	NA	Y	N	NA	NA	Good
Faraj (38)	Y	Y	Y	Y	Y	NA	NA	NA	NA	NA	Y	N	NA	NA	Good
Grijalva et al. (39)	Y	Y	Y	Y	Y	NA	NA	NA	NA	NA	Y	N	NA	NA	Good
Haque et al. (40)	Y	Y	Y	Y	NI	NA	NA	NA	NA	NA	Y	N	NA	NA	Fair
Hasib et al. (41)	Y	Y	Y	Y	NI	NA	NA	NA	NA	NA	Y	N	NA	NA	Fair
Hein et al. (42)	Y	Y	Y	Y	Y	NA	NA	NA	NA	NA	Y	N	NA	NA	Good
Hoddinott et al. (43)	Y	Y	Y	Y	NI	NA	NA	NA	NA	NA	Y	N	NA	NA	Fair
Idowu et al. (15)	**Y**	**Y**	**Y**	**Y**	**Y**	**NA**	**NA**	**NA**	**NA**	**NA**	**Y**	**N**	**NA**	**NA**	**Good**
Jayatissa et al. (44)	Y	Y	Y	Y	Y	NA	NA	NA	NA	NA	Y	N	NA	NA	Good
Jemal et al. (45)	Y	Y	Y	Y	Y	NA	NA	NA	NA	NA	Y	N	NA	NA	Good
Komasi (46)	Y	Y	Y	Y	NI	NA	NA	NA	NA	NA	Y	N	NA	NA	Fair
Mandre et al. (47)	Y	Y	Y	Y	Y	NA	NA	NA	NA	NA	N	N	NA	NA	Fair
Centers for Disease Control (CDC) (48)	Y	Y	Y	Y	NI	NA	NA	NA	NA	NA	Y	N	NA	NA	Fair
Nwagboso (49)	Y	Y	Y	Y	Y	NA	NA	NA	NA	NA	Y	N	NA	NA	Good
Olwedo et al. (50)	Y	Y	Y	Y	CD	NA	NA	NA	NA	NA	Y	N	NA	NA	Fair
Praditsorn et al. (51)	Y	Y	Y	Y	Y	NA	NA	NA	NA	NA	Y	N	NA	NA	Good
Pernitez-Agan et al. (52)	Y	Y	Y	Y	NI	NA	NA	NA	NA	NA	Y	N	NA	NA	Fair
Smock et al. (13)	Y	Y	Y	Y	NI	NA	NA	NA	NA	NA	Y	N	NA	NA	Fair
Vakos et al. (53)	Y	Y	Y	Y	NI	NA	NA	NA	NA	NA	Y	N	NA	NA	Fair
Walpole et al. (54)	Y	Y	Y	Y	NI	NA	NA	NA	NA	NA	Y	N	NA	NA	Fair

Y, yes; N, No; NA, not applicable; CD, cannot determine; NI, no information.

Q1: Was the research question or objective in this paper clearly stated?

Q2: Was the study population clearly specified and defined?

Q3: Was the participation rate of eligible persons at least 50%?

Q4: Were all the subjects selected or recruited from the same or similar populations (including the same time period)? Were inclusion and exclusion criteria for being in the study prespecified and applied uniformly to all participants?

Q5: Was a sample size justification, power description, or variance and effect estimates provided?

Q6: For the analyses in this paper, were the exposure(s) of interest measured prior to the outcome(s) being measured?

Q7: Was the timeframe sufficient so that one could reasonably expect to see an association between exposure and outcome if it existed?

Q8: For exposures that can vary in amount or level, did the study examine different levels of the exposure as related to the outcome (e.g., categories of exposure, or exposure measured as continuous variable)?

Q9: Were the exposure measures (independent variables) clearly defined, valid, reliable, and implemented consistently across all study participants?

Q10: Was the exposure(s) assessed more than once over time?

Q11: Were the outcome measures (dependent variables) clearly defined, valid, reliable, and implemented consistently across all study participants?

Q12: Were the outcome assessors blinded to the exposure status of participants?

Q13: Was loss to follow-up after baseline 20% or less?

Q14: Were key potential confounding variables measured and adjusted statistically for their impact on the relationship between exposure(s) and outcome(s)?

#### Baseline features of the included studies

The baseline characteristics of each article were analyzed and summarized (participants, study design, country of origin of refugees, and outcome) in Table 3. Of the 30 studies, 29 were cross-sectional (14–16, 29–54), while one record-based study was found (13). The study period ranged from 2005 to 2022, with 22 studies conducted among the refugee population and eight studies conducted among internally displaced population. In terms of geographical distribution, the majority (12 out of 30) of the refugees originated from the African region (15, 32, 34–37, 39, 45, 47, 49, 50), followed by the Eastern Mediterranean Region (eight out of 30) (16, 29–31, 33, 52–54) and South-East Asian region of WHO member states (nine out of 30) (14, 38, 40–44, 48, 51). One study was multicountry research (13), where the refugee population included were from Africa, South, East and Central Asia, and the Pacific African countries. In eight studies, participants were internally displaced, out of which four studies had population groups from the African region (Nigeria, Uganda, Burkina Faso, and Cameroon) (15, 32, 34, 50), and four studies had population groups from South-East Asian (Srilanka, Pakistan, Bangladesh, and Myanmar) (14, 40, 42, 44). Overall, 30 studies, including 31,565 under-five refugee and internally displaced children, were found eligible for the meta-analysis. The range of sample size was 100 (41) to 14,552 (52). Overall, 50.4% of study participants were men and 49.6% were women. The children in the review ranged from newborn to 5 years of age. There was a varying proportion of under-five refugee children with stunting ranging from 9% in Syrian refugees residing in Lebanon (Western Asia) (30) to 59.4% in the internally displaced population residing in Myanmar (42) (Table 3).

**Table 3:** Baseline characteristics of refugee/IPD children with stunting (*N* = 30 studies).

**References**	**Residing country of refugee/IDPs**	**Origin country of refugee**	**Study design**	**Sample**	**Stunting prevalence**	**Age**	**Men (%)**	**Women (%)**
Praditsorn et al. (51)	Thailand Myanmar Border	Mynmar	Cross-sectional study	2,702	28.8%	6–59 months (mean: 30 ± 15 month)	52.1%	47.9%
Grijalva-Eternod et al. (39)	Algeria	Western Saharan region	Cross-Sectional study	1,608	29.1%	6–59 months	51.4%	48.6%
El Kishawi et al. (16)	Palestine	Palestine territory	Cross-Sectional study	357	19.60%	2–5 years (mean: (39.58 ± 10.74 month)	52.7%	47.3%
Ali et al. (14)	Pakistan	Internally displaced	Cross-Sectional study	446	12.5%	6–59 months	45.1%	54.9%
Idowu et al. (15)	Nigeria	Internally displaced	Cross-sectional study	317	53.9%	0–59 months (median: 24 months)	50.8%	49.2%
Walpole et al. (54)	Greece	Syria	Cross-Sectional study	114	16.7%	< 5 years	50.8%	49.2%
Abou-Rizk et al. (30)	Lebanon	Syria	Cross-Sectional study	432	9%	< 5 years (mean: 16.7 ± 14.3 months)	52.5%	47.5%
Smock et al. (13)	Massachusetts	Multicountry	Record based study	1,561	10%	Under 5 years	51.1%	48.8%
Abukishk et al. (31)	Jordan	Palestine	Cross-Sectional study	367	22.9%	Under 5 years	49.6%	50.4%
Abdeen et al. (29)	Palestine	Palestine territory	Cross-Sectional study	1,331	12.4%	6–59 months	51.4%	48.6%
Olwedo et al. (50)	Uganda	Internally displaced	Cross-Sectional study	672	52.4%	3–59 months	1.2%	98.8%
Centers for Disease Control (CDC) (48)	Nepal	Bhutan	Cross-Sectional study	497	26.9%	6–59 months	–	–
Hoddinott et al. (43)	Bangladesh	Mynmar	Cross-Sectional study	523	33.4%	6–23 months (mean: 15.5 ± 4.7 months)	47.8%	52.2%
Jayatissa et al. (44)	Sri Lanka	Internally displaced	Cross-Sectional study	878	20.2%	Under 5 years	49.1%	50.9%
Bougma et al. (34)	Burkina Faso	Internally displaced	Cross-Sectional study	205	45.9%	Under 5 years	52.2%	47.8%
Haque et al. (40)	Bangladesh	Internally displaced	Cross-Sectional study	387	45%	3–5 years (mean: 4.10 ± 0.84)	–	–
Hein et al. (42)	Myanmar	Internally displaced	Cross-Sectional study	320	59.4%	6–59 months	56.9%	43.1%
Pernitez-Agan et al. (52)	Turkey, Lebanon, Jordan, Iraq, Egypt	Syria	Cross-Sectional study	14,552	9.1%	6–59 months	51.6%	48.4%
Hasib et al. (41)	Bangladesh	Myanmar	Cross-Sectional study	100	41%	0–5 years	–	–
Komasi (46)	Ghanna	La Cote D‘Ivoire	Cross-Sectional study	150	18%	6–59 months	54%	46%
Faraj (38)	Thailand	Burma	Cross-Sectional study	540	46.5%	6–59 months	51.1%	48.9%
Mandre et al. (47)	Uganda	Sudan	Cross-Sectional study	340	24.7%	6–59 months	–	–
Vakos et al. (53)	Jordan	Syria	Cross-Sectional study	165	13.9%	0–59 months	–	–
Bilukha et al. (33)	Jordan	Syria	Cross-Sectional study	327	17%	6–59 months	–	–
Faine et al. (37)	Cameroon	Nigeria	Cross-Sectional study	366	22.4%	6–59 months	53%	47%
Jemal and Haidar (45)	Ethiopia	Somalia	Cross-Sectional study	671	27.6%	6–59 months	51.7%	48.3%
Brhane (35)	Ethiopia	Eritrea	Cross-Sectional study	471	37%	6–59 months	50.74%	49.25%
Nwagboso (49)	Namibia	Angola	Cross-Sectional study	574	41.46%	6–59 months	44.94%	55.06%
Ejigu et al. (36)	Ethiopia	Somalia, Sudan, Eritrea	Cross-Sectional study	367	18.8%	6–59 months	51.5%	48.5%
Akeh et al. (32)	Cameroon	Internally displaced	Cross-Sectional study	395	22.1%	6–59 months 38.4 ± 17.7 months	59%	41%

### Pooled estimate of stunting

**Figure 2:**
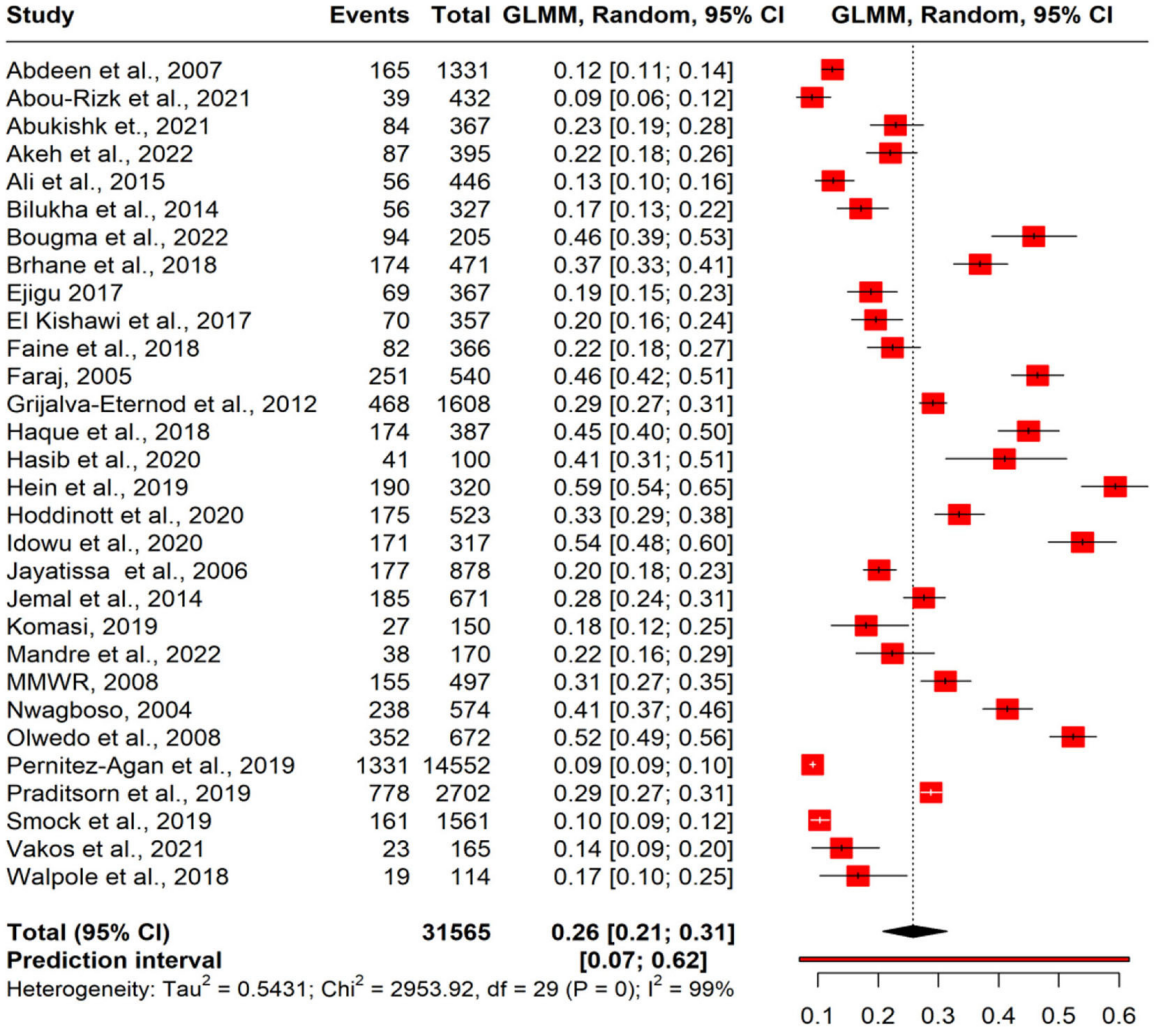
Forest plot of pooled magnitude of stunting in refugee children. GLMM, generalized linear mixed effects model.

**Figure 3:**
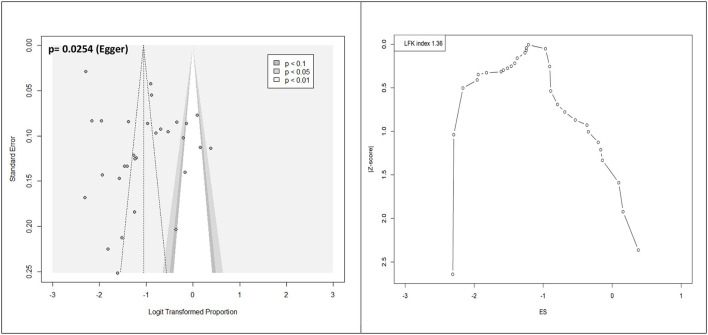
Publication bias assessed by contour-enhanced funnel plot and Doi plot.

**Figure 4:**
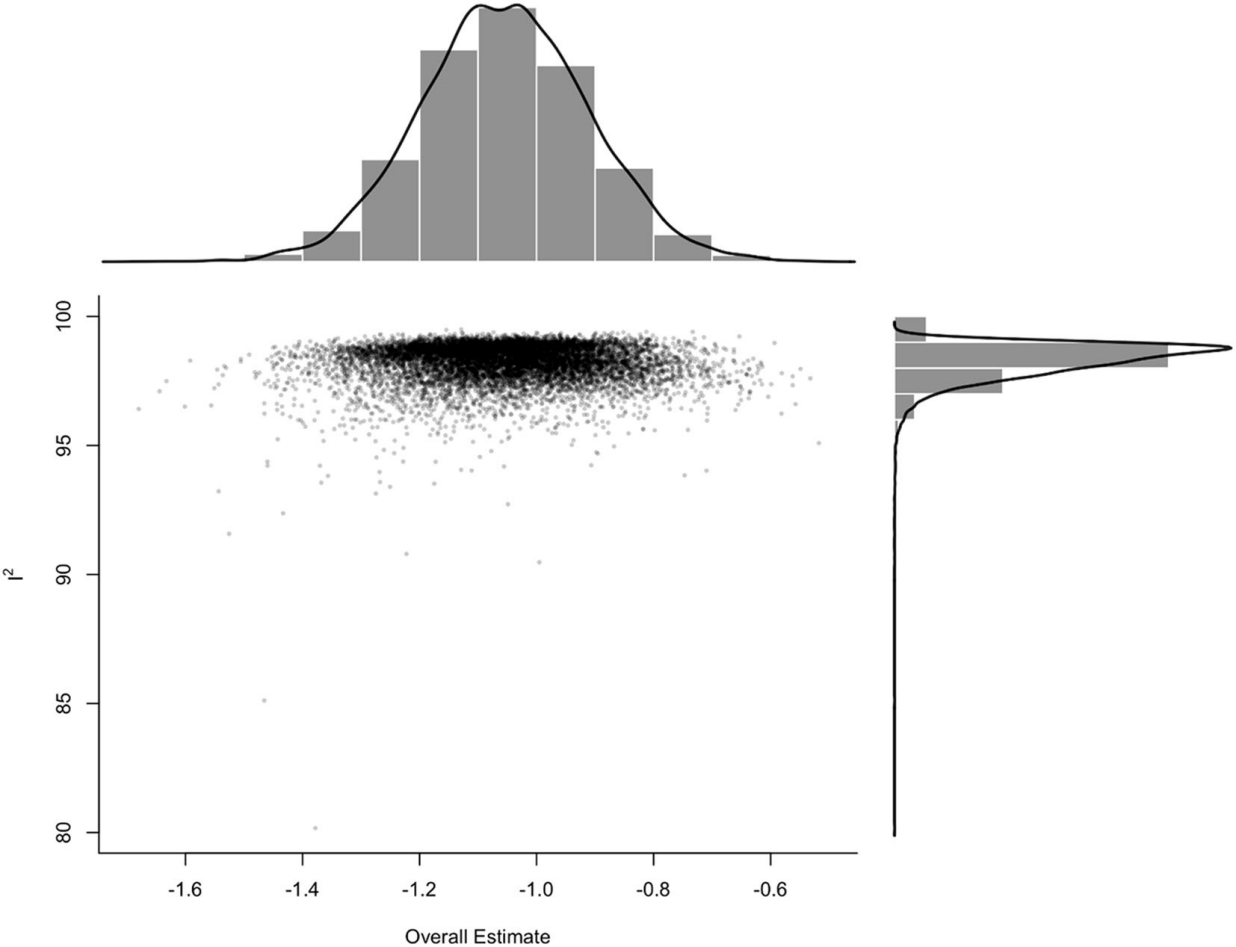
Graphic display of heterogeneity (GOSH) plot analysis.

**Figure 5:**
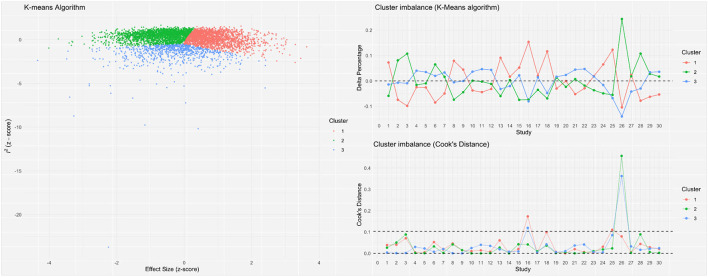
*K*-means GOSH plot analysis.

**Figure 6:**
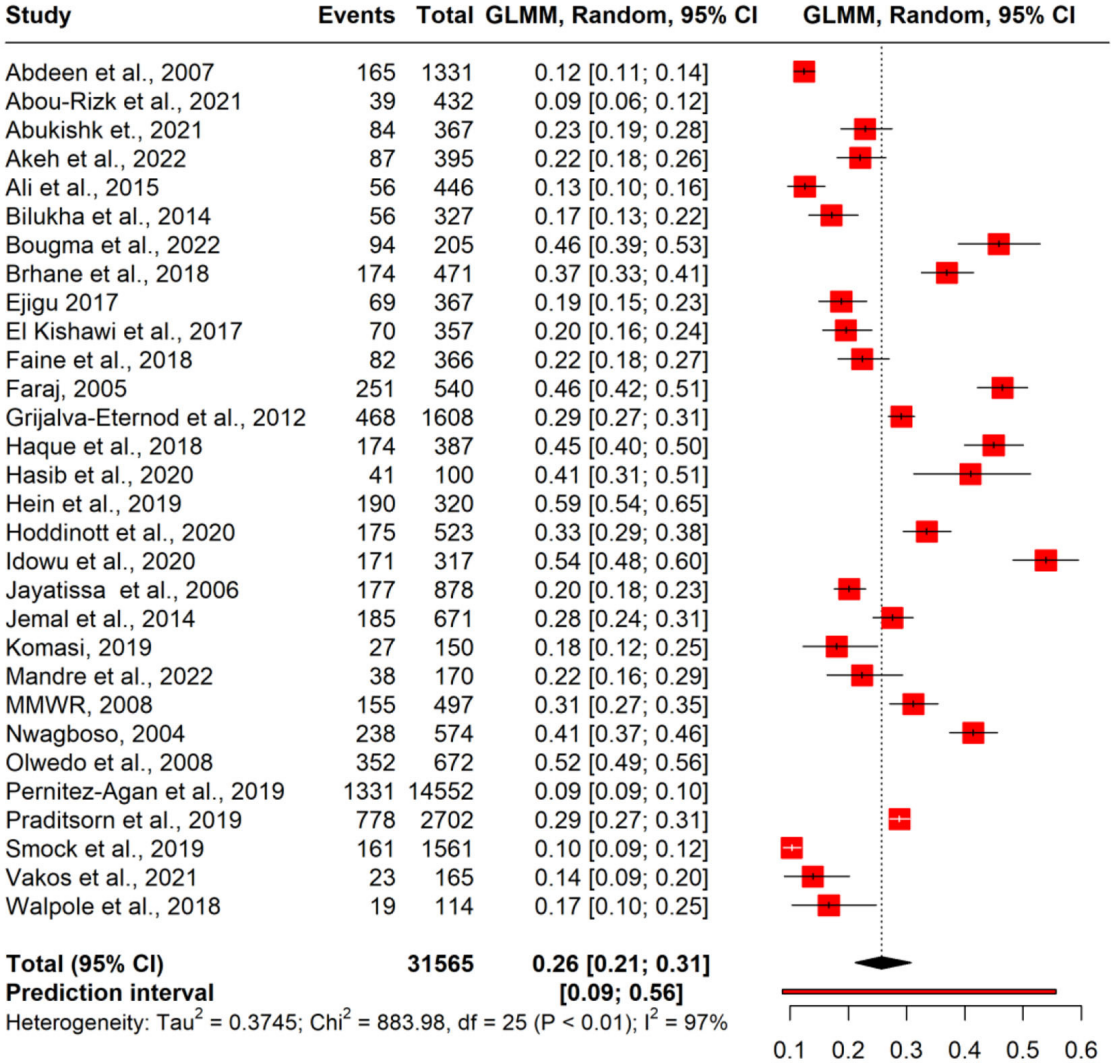
Forest plot after removal of potential outlier through GOSH plot analysis.

A meta-analysis was performed to evaluate the prevalence of stunting among 31,565 under-five refugee and internally displaced children, among whom 5,930 had stunting. The effect size (pooled prevalence) of stunting in under-five refugee and internally displaced children was 26% [95% confidence interval (CI), 21–31]. The prediction interval was found to be between 7 and 62%. The mean effect size of comparable studies would fall anywhere in this prediction interval (Figure 2). High heterogeneity was found in the current meta-analysis (*I*^2^ = 99%; *p* = < 0.001), reflecting variance in true effects rather than sampling error. Hence, a random-effects model was applied. The contour-enhanced funnel plot showed an asymmetrical funnel with Egger's statistics *p*-value of 0.0254. The LFK index was 1.36 in the Doi plot, which revealed a small study effect or publication bias (Figure 3). The Baujat and diagnostic plots were made to identify studies contributing to heterogeneity (Supplementary Figures 1, 2). Leave-one-out analysis revealed no significant change in the pooled estimate or the heterogeneity (Supplementary Figure 3). GOSH plot analysis (*K*-means, DBSCAN, and Gaussian) revealed that the studies by Abou-Risk et al., Hein et al., Pernitez-Agan et al., and Olwedo et al. were the potential outliers, and the pooled analysis was conducted after removing the outlier studies (Figures 4, 5; Supplementary Figures S4–S9). The forest plot was made after the removal of potential outliers through GOSH plot analysis, where it was found that the pooled estimate of stunting was the same- 26% (CI- 21 to 31) but the heterogeneity had decreased to 97% (Figure 6).

#### Subgroup analysis

Subgroup analysis conducted on the basis of type of children revealed that internally displaced children (IDP group) had a higher prevalence of stunting (37%) when compared to the refugee children (22%) (Table 4). According to the WHO regions, refugees from the South-East Asian region (SEAR) had the highest prevalence of stunting (34%) when compared with African countries (32%) and Eastern Mediterranean Region (EMR) (14%) (Table 4). However, heterogeneity remained high between the studies within the subgroups (95–99%).

**Table 4:** Subgroup analysis of the studies reporting the stunting based on refugee population and internally displaced person.

**Factors**	**No. of studies**	**Estimate (95% CI)**	***p***	***I* ^2^**	**P subgroup**
WHO Regions^*^					< 0.01
Africa	12	32 (24–40)	< 0.01	97%	
South-East Asian region	5	34 (24–46)	< 0.01	97%	
Eastern Mediterranean region	8	14 (11–19)	< 0.01	95%	
Multi Region	1	10 (9–12)	< 0.01		
IDP/Refugee					0.02
Internally displaced person	8	37 (23–53)	< 0.01	98%	
Refugee	22	22 (18–28)	< 0.01	99%	

A mixed-effects model was applied to the subgroup meta-analysis.

^*^One multi-region study (13).

## Discussion

The pooled prevalence of stunting has been estimated to be 26% among the under-five children of the refugees and internally displaced population in the index analysis. This is marginally higher than the global stunting rates reported among the under-five children (22%) (55). However, while subgrouping, children belonging to IDPs had a significantly higher prevalence of stunting (37%) than the refugees (22%), indicating higher vulnerability among the IDPs. This might be due to the impact of the socioeconomic capability of the host country in providing aid and provisions for the refugees. In contrast, since the IDPs have moved to a different place within the same country, the country's capacity remains the same, while on the other hand, the environment milieu has been changed for such people. The IDPs included in the present analysis were from either low-income or low-middle-income countries in Asia and Africa, thus restricting the economic capability of the country to respond adequately to the IDPs. The stunting rate was less in countries with high human development index (HDI) and vice versa, which means there is a linear relationship between HDI and stunting rate (56).

Across the world, 59.1 million people are internally displaced (21). The vulnerability of the IDPs has tended to remain high compared to the refugees owing to the IDP camps located close to the conflicts or, at times, trapped within the conflict zones (57). Research among this vulnerable section-IDPs, to bring out the potential factors has also been lacking (58). One of the potential reasons for the high vulnerability among IDPs might be due to non-compliance of the respective country/nation states to the international norms on the IDPs (59), and it is seen as an internal issue by the country, with limited role or part allowed for the international community to play. Analytical and qualitative studies assessing the determinants of nutrition between the refugees and IDPs may shed further clarity on the variations.

Regarding the regional variation, children from refugees and IDPs from the SEAR had the highest stunting rate (34%), followed by the African region (32%), which was higher than the global rate. Refugee children had a 50% higher stunting rate than their counterparts among the general population of the African region (31%) (59). The intersection of the African region and the attribute of internal displacement has revealed high stunting ranging upto 54% (15). This adverse intersection needs to be further evaluated for the underlying causes and addressed adequately. South-East Asian countries reported a stunting rate of 34%, which was slightly higher than the rates prevailing among the general population (27%) (60). IDPs from Myanmar reported the highest prevalence of stunting among the included children (59%) (42). Lack of dietary diversity has been attributed as a potential factor for this high rate of stunting (42). Children of the Rohingyas from Myanmar, currently in Bangladesh, had a stunting rate of 33.4% (43), which was also the highest among the Asian countries included in the analysis. Rohingyas are labeled the “most persecuted minority in the world” by the United Nations and, consequently, the most vulnerable population for poor health outcomes (61). None of the Rohingyas who moved into Bangladesh in or after 2017 were given refugee status (43), but are called “Forcibly Displaced Myanmar Nationals” (62).

This is the first study to estimate the pooled prevalence of stunting among under-five refugee and internally displaced children worldwide. Objective tools were used to assess and report the quality of the studies included in the meta-analysis. A better measure of publication bias, the Doi plot, was used to assess the publication bias between the studies. High heterogeneity and the potential publication bias are the major limitations. Variation in the field application of the tools to measure anthropometry is also a limitation, contributing to the high heterogeneity. The authors have explored the heterogeneity by means of subgroup analysis and sensitivity analysis. GHOSH plots and diagnostic tests were applied to identify outliers, and sensitivity analysis was conducted. Although heterogeneity could not be reduced, it revealed a differential pattern of stunting prevalence between the type of refugee/IDP and the geographical origin. The persistence of high heterogeneity indicates that racial, ethnic, and socio-cultural factors might have more impact on the nutritional status.

## Conclusion

The stunting rate among the under-five children of refugees and IDPs is 24%, with a higher prevalence among the IDPs (32%). Geographically, refugees and IDP children from the African region, and ethnically, the Rohingya children are the most vulnerable and stunted. Further research on the determinants of the nutrition status of the African IDPs and Rohingyas needs to be conducted. Implementation of interventions to address the disproportionately higher stunting among the children of IDPs, Rohingyas, and the African region might improve the nutritional status of these marginalized groups.

## Data availability statement

The original contributions presented in the study are included in the article/Supplementary material, further inquiries can be directed to the corresponding authors.

## Author contributions

PC: Conceptualization, Data curation, Investigation, Methodology, Visualization, Writing—original draft. BP: Conceptualization, Data curation, Formal analysis, Methodology, Resources, Validation, Visualization, Writing—original draft. AM: Conceptualization, Data curation, Investigation, Methodology, Writing—original draft. AG: Conceptualization, Formal analysis, Methodology, Project administration, Writing—original draft, Writing—review & editing. SM: Conceptualization, Formal analysis, Methodology, Writing—original draft. NS: Conceptualization, Data curation, Methodology, Writing—original draft. SB: Conceptualization, Data curation, Methodology, Writing—original draft. PS: Conceptualization, Data curation, Methodology, Writing—original draft. MS: Conceptualization, Data curation, Formal analysis, Software, Writing—original draft. LT: Data curation, Formal analysis, Methodology, Writing—original draft. SR: Conceptualization, Formal analysis, Project administration, Supervision, Writing—original draft. RS: Conceptualization, Formal analysis, Project administration, Supervision, Writing—original draft. MK: Data curation, Methodology, Resources, Writing—review & editing. SG: Conceptualization, Methodology, Resources, Writing—review & editing. QZ: Data curation, Methodology, Resources, Writing—review & editing. AA-A: Data curation, Methodology, Software, Writing—review & editing. HA: Conceptualization, Data curation, Funding acquisition, Methodology, Project administration, Writing—original draft.

## References

[1] World Health Organization Department of Nutrition for Health and Development. WHO Child Growth Standards: Length/Height-for-Age, Weight-for-Age, Weight-for-Length, Weight-for-Height and Body Mass Index-for-Age: Methods and Development. Geneva (2006).

[2] Black RE Allen LH Bhutta ZA Caulfield LE de Onis M Ezzati M . Maternal and child undernutrition: global and regional exposures and health consequences. Lancet. (2008) 371:243–60. 10.1016/S0140-6736(07)61690-0 18207566

[3] UNICEF WHO WBG. Levels and Trends in Child Malnutrition: UNICEF/WHO/World Bank Group Joint Child Malnutrition Estimates: Key Findings of the 2020 Edition. Geneva (2020).

[4] Victora CG Adair L Fall C Hallal PC Martorell R Richter L . Maternal and child undernutrition: consequences for adult health and human capital. Lancet. (2008) 371:340–57. 10.1016/S0140-6736(07)61692-4 18206223 PMC2258311

[5] United Nations High Commissioner for Refugees. Who is a Refugee, Who is an Internally Displaced Person. Washington, DC: UN Refug Agency (2021).

[6] United Nations High Commissioner for Refugees. Figures at a Glance, UNHCR Global Trends 2021. Washington, DC: UN Refug Agency (2022).

[7] Gushulak BD MacPherson DW. Health aspects of the pre-departure phase of migration. PLoS Med. (2011) 8:e1001035. 10.1371/journal.pmed.1001035 21629679 PMC3101199

[8] Gushulak BD Pottie K Roberts JH Torres S DesMeules M. Migration and health in Canada: health in the global village. Cmaj. (2011) 183:E952–8. 10.1503/cmaj.090287 20584934 PMC3168671

[9] Jaeger FN Hossain M Kiss L Zimmerman C. The health of migrant children in Switzerland. Int J Public Health. (2012) 57:659–71. 10.1007/s00038-012-0375-8 22699954

[10] European Centre for Disease Prevention and Control. Assessing the Burden of Key Infectious Diseases Affecting Migrant Populations in the EU/EEA. Solna: Stock ECDC (2014).

[11] Hjern A Koçtürk-Runefors T Jeppson O Tegelman R Höjer B Adlercreutz H. Health and nutrition in newly resettled refugee children from Chile and the Middle East. Acta Paediatr Scand. (1991) 80:859–67. 10.1111/j.1651-2227.1991.tb11961.x 1957607

[12] Modgil G Williams B Oakley G Burren CP. High prevalence of Somali population in children presenting with vitamin D deficiency in the UK. Arch Dis Child. (2010) 95:568–9. 10.1136/adc.2010.187435 20522462

[13] Smock L Nguyen T Metallinos-Katsaras E Magge H Cochran J Geltman PL. Refugee children's participation in the women, infants, and children supplemental nutrition (WIC) program in Massachusetts, 1998-2010. J Public Heal Manag Pract. (2019) 25:69–77. 10.1097/PHH.0000000000000789 29672357

[14] Ali W Ayub A Hussain H. Prevalence and associated risk factors of under nutrition among children aged 6 to 59 months in internally displaced persons of Jalozai Camp, District Nowshera, Khyber Pakhtunkhwa. J Ayub Med Coll Abbottabad. (2015) 27:556–9. 26721006

[15] Idowu SO Akindolire AE Adebayo BE Adebayo AM Ariyo O. Determinants of anthropometric characteristics of under-five children in internally displaced persons camps in Abuja municipal area council, Abuja, Nigeria. Pan Afr Med J. (2020) 36:1–12. 10.11604/pamj.2020.36.313.21221 33193967 PMC7603821

[16] El Kishawi RR Soo KL Abed YA Muda WAMW. Prevalence and associated factors influencing stunting in children aged 2-5years in the Gaza Strip-Palestine: a cross-sectional study. BMC Pediatr. (2017) 17:1–7. 10.1186/s12887-017-0957-y 29268788 PMC5740756

[17] Skinner A Tester-Jones MC Carrieri D. Undernutrition among children living in refugee camps: a systematic review of prevalence. BMJ Open. (2023) 13:e070246. 10.1136/bmjopen-2022-070246 37321810 PMC10277121

[18] Ankomah A Byaruhanga J Woolley E Boamah S Akombi-Inyang B. Double burden of malnutrition among migrants and refugees in developed countries: a mixed-methods systematic review. PLoS ONE. (2022) 17:e0273382. 10.1371/journal.pone.0273382 35981085 PMC9387835

[19] Page MJ McKenzie JE Bossuyt PM Boutron I Hoffmann TC Mulrow CD . The PRISMA 2020 statement: an updated guideline for reporting systematic reviews. Int J Surg. (2021) 88:105906. 10.1016/j.ijsu.2021.105906 33789826

[20] United Nations High Commissioner for Refugees. Convention and Protocol Relating to the Status of Refugees. Geneva (2011). 12344222

[21] Internal Displacement Monitoring Centre. Global Report on Internal Displacement. Geneva (2022).

[22] National Institutes of Health (NIH) National Heart and Lung and Blood Institute. Study Quality Assessment Tools. Bethesda (2021).

[23] Thompson SG Smith TC Sharp SJ. Investigating underlying risk as a source of heterogeneity in meta-analysis. Stat Med. (1997) 16:2741–58. 10.1002/(SICI)1097-0258(19971215)16:23<2741::AID-SIM703>3.0.CO;2-0 9421873

[24] Chandler J Higgins JP Deeks JJ Davenport CM. Handbook for systematic reviews of interventions. Cochrane Handb Syst Rev Interv. (2017) 520:1–11.

[25] Egger M Smith GD Schneider M Minder C. Bias in meta-analysis detected by a simple, graphical test. Bmj. (1997) 315:629–34. 10.1136/bmj.315.7109.629 9310563 PMC2127453

[26] Furuya-Kanamori L Barendregt JJ Doi SAR. A new improved graphical and quantitative method for detecting bias in meta-analysis. Int J Evid Based Healthc. (2018) 16:195–203. 10.1097/XEB.0000000000000141 29621038

[27] Gandhi AP Shamim MA Padhi BK. Steps in undertaking meta-analysis and addressing heterogeneity in meta-analysis. Evid. (2023) 1:44–59.

[28] Shamim MA Gandhi AP Dwivedi P Padhi BK. How to perform meta-analysis in R: a simple yet comprehensive guide. Evid. (2023) 1:60–80.

[29] Abdeen Z Greenough PG Chandran A Qasrawi R. Assessment of the nutritional status of preschool-age children during the Second Intifada in Palestine. FOOD Nutr Bull. (2007) 28:274–82. 10.1177/156482650702800303 17974360

[30] Abou-Rizk J Jeremias T Nasreddine L Jomaa L Hwalla N Tamim H Frank J Scherbaum V. Anemia and nutritional status of syrian refugee mothers and their children under five years in greater Beirut, Lebanon. Int J Environ Res Public Health. (2021) 18:6894. 10.3390/ijerph18136894 34199032 PMC8297067

[31] Abukishk N Gilbert H Seita A Mukherjee J Rohloff PJ. Under-five malnutrition among Palestine refugee children living in camps in Jordan: a mixed-methods study. BMJ Glob Heal. (2021) 6:e005577. 10.1136/bmjgh-2021-005577 34348932 PMC8340287

[32] Akeh ML Tendongfor N Nchung AJ Chipili G Mbhenyane X Tambe AB. Magnitude and predictors of malnutrition among internally displaced persons' children 6–59 months in Bamenda Health District of Cameroon: a community-based cross-sectional study. Nutr Health. (2022) 1–8. 10.1177/02601060221132134 36237133

[33] Bilukha OO Jayasekaran D Burton A Faender G King'ori J Amiri M . Nutritional status of women and child refugees from Syria-Jordan, April-May 2014. MMWR Morb Mortal Wkly Rep. (2014) 63:638–9. 25055188 PMC5779421

[34] Bougma S Hama-Ba F Garanet F Savadogo A. Nutritional status of children under five years of age among internally displaced populations and non-displaced in Burkina Faso. J Food Nutr Res. (2022) 10:449–58. 10.12691/jfnr-10-7-2

[35] Brhane H. Prevalence and associated factors of acute malnutrition among 6-59 month children in Adi-Harush and Hitsats Refugee Camps in Tigray Region Northern Ethiopia, 2017. Am J Life Sci. (2018) 6:57. 10.11648/j.ajls.20180605.11

[36] Ejigu B Legesse TG Chercos DH. Prevalence and associated factors of malnutrition among children aged 6–59 months in Addi Harush Eritrean Refugees camp, Tigray Region, North Ethiopia. J Pharm Nutr Sci. (2017) 7:164–71. 10.6000/1927-5951.2017.07.04.3

[37] Faine D Fon PN Mbuagbaw L Tegang SC Yobo ABE Chiabi A. Anthropometric measurements in children 6-59 months old in the Minawao refugee camp, in the far North Region of Cameroon. Clin Res Pediatr. (2018) 1:1–9.

[38] Faraj N. Nutritional Status of Under Five Year Old Burmese Refugee Children in Thailand. Honolulu, HI: University of Hawaii at Manoa (2005).

[39] Grijalva-Eternod CS Wells JCK Cortina-Borja M Salse-Ubach N Tondeur MC Dolan C . The double burden of obesity and malnutrition in a protracted emergency setting: a cross-sectional study of Western Sahara refugees. PLoS Med. (2012) 9:e1001320. 10.1371/journal.pmed.1001320 23055833 PMC3462761

[40] Haque MM Islam K. Socio-economic condition, dietary pattern and nutritional status of pre-school children among settlers and ethnic communities in Bandarban District of Bangladesh. Arch Community Fam Med. (2019) 2:8–19. 10.22259/2638-4787.0202002

[41] Hasib M Hassan MN Hasan M Khan MSI. Effect of nutritional status on Rohingya under-five children in Bangladesh. Int J Public Heal Sci. (2020) 9:358–63. 10.11591/ijphs.v9i4.20546

[42] Hein AK Hong SA Puckpinyo A Tejativaddhana P. Dietary diversity, social support and stunting among children aged 6–59 months in an internally displaced persons camp in Kayin state, Myanmar. Clin Nutr Res. (2019) 8:307–17. 10.7762/cnr.2019.8.4.307 31720256 PMC6826059

[43] Hoddinott J Dorosh P Filipski M Rosenbach G Tiburcio E. Food transfers, electronic food vouchers and child nutritional status among Rohingya children living in Bangladesh. PLoS ONE. (2020) 15:0230457. 10.1371/journal.pone.0230457 32348313 PMC7190090

[44] Jayatissa R Bekele A Piyasena CL Mahamithawa S. Assessment of nutritional status of children under five years of age, pregnant women, and lactating women living in relief camps after the tsunami in Sri Lanka. Food Nutr Bull. (2006) 27:144–52. 10.1177/156482650602700205 16786980

[45] Jemal Y Haidar J. Chronic malnutrition and its determinants among refugee children: evidence from refugee camp of Ethiopia. East Afr J Public Health. (2014) 11:816–22.

[46] Komasi S. Feeding Practices and Nutritional Status of Children in Ampain Refugee Camp, Ghana. Ghanna: University of Cape Coast (2019).

[47] Mandre J Kaindi DWM Kogi-Makau W. Nutrition status of refugee and host-country children: negotiating for equal distribution of relief food during emergencies in Uganda. J Immigr Minor Heal. (2022) 24:1387–97. 10.1007/s10903-022-01354-4 35347536

[48] Centers for Disease Control and Prevention (CDC). Malnutrition and Micronutrient Deficiencies Among Bhutanese Refugee Children–Nepal, 2007. Clifton Road Atlanta, GA (2008). p. 370–3. 18401331

[49] Nwagboso GC. An Evaluation of the Nutritional Status of Refugee Children in Namibia. University of the Western Cape, South Africa (2004).

[50] Olwedo MA Mworozi E Bachou H Orach CG. Factors associated with malnutrition among children in internally displaced person's camps, northern Uganda. Afr Health Sci. (2008) 8:244–52. 20589132 PMC2887019

[51] Praditsorn P Churak P Wimonpeerapattana W Moore T Bovill M. Prevalence of undernutrition and associated factors among children 6 to 59 months of age in refugee camps along Thailand-Myanmar border. Southeast Asian J Trop Med Public Health. (2019) 50:372–82.

[52] Pernitez-Agan S Wickramage K Yen C Dawson-Hahn E Mitchell T Zenner D. Nutritional profile of Syrian refugee children before resettlement. Confl Health. (2019) 13:22. 10.1186/s13031-019-0208-y 31171934 PMC6549318

[53] Vakos A Khalil N Kumar A Menezes L Ahson M. Assessment of growth in pediatric syrian refugee populations in Jordan. Avicenna J Med. (2021) 11:167–71. 10.1055/s-0041-1736544 34881199 PMC8648405

[54] Walpole SC Abbara A Gunst M Harkensee C. Cross-sectional growth assessment of children in four refugee camps in Northern Greece. Public Health. (2018) 162:147–52. 10.1016/j.puhe.2018.05.004 30075409

[55] World Health Organisation. Joint Child Malnutrition Estimates. Geneva: WHO (2021). p. 5–10.

[56] Joulaei H Keshani P Ashourpour M Bemani P Amiri S Rahimi J . The prevalence of stunting among children and adolescents living in the Middle East and North Africa region (MENA): A systematic review and meta-analysis. J Glob Health. (2021) 11. 10.7189/jogh.11.04070 35003712 PMC8711751

[57] United Nation Human Rights. About internally displaced persons [Internet]. (2023). Available online at: https://www.ohchr.org/en/special-procedures/sr-internally-displaced-persons/about-internally-displaced-persons

[58] Orendain DJA Djalante R. Ignored and invisible: internally displaced persons (IDPs) in the face of COVID-19 pandemic. Sustain Sci. (2021) 16:337–40. 10.1007/s11625-020-00848-0 32837575 PMC7406698

[59] Global Nutrition Report. Country Nutrtion Profiles. Bristol: Development Initiatives (2022).

[60] Global Nutrition Report. Country Nutrition Profiles - Ecuador. Bristol: Development Initiatives (2022). p. 1–17.

[61] USA for UNHCR. Rohingya Refugee Crisis: Supporting the Stateless Minority Fleeing Myanmar [Internet]. (2023). Available online at: https://www.unrefugees.org/emergencies/rohingya/

[62] Ismail M Hussain MF Abdullah al Hasan M Kamal AHMM Rahman M Hasan MJ. Health problems among Forcibly Displaced Myanmar Nationals (FDMNs) admitted to the Medicine ward of Cox's Bazar Medical College Hospital. J Migr Heal. (2022) 6:100123. 10.1016/j.jmh.2022.100123 35694421 PMC9184555

